# Predicting the diagnostic efficacy of trio-based whole exome sequencing in children with low-function autism spectrum disorders: a multicenter study

**DOI:** 10.3389/fneur.2025.1597588

**Published:** 2025-10-07

**Authors:** Ruohao Wu, Xiangyang Luo, Zhanwen He, Zhe Meng, Wenting Tang, Liyang Liang

**Affiliations:** ^1^Department of Children’s Neuro-endocrinology, Sun Yat-sen Memorial Hospital, Sun Yat-sen University, Guangzhou, Guangdong, China; ^2^Children’s Medical Center, Sun Yat-sen Memorial Hospital, Sun Yat-sen University, Guangzhou, Guangdong, China; ^3^Weierkang Children’s Rehabilitation Center, Guangzhou, Guangdong, China; ^4^Department of Research and Molecular Diagnostics, Sun Yat-sen University Cancer Center, Sun Yat-sen University, Guangzhou, Guangdong, China

**Keywords:** low-function autism spectrum disorders, neurodevelopmental/neurological comorbidities, trio-based whole-exome sequencing, diagnostic rate, phenotype-driven, nomogram, genetic abnormalities

## Abstract

**Background:**

Although significant progress has been made in trio-based whole-exome sequencing (trio-WES) that enables the detection of exon-level variants, the diagnostic effectiveness of empirical and unselected use of trio-WES in children with low-function autism spectrum disorders (LF-ASDs) remains unsatisfactory. Thus, the identification of an appropriate approach for predicting the diagnostic efficacy of trio-WES at the pre-diagnosis stage is essential for implementing individualized diagnosis for children with LF-ASDs.

**Methods:**

A total of 168 LF-ASDs patients who underwent trio-WES at Sun Yat-sen Memorial Hospital from September 2016 to December 2022 were enrolled as the training set. Additionally, 58 LF-ASDs patients who received trio-WES at Weierkang Children’s Rehabilitation Center between January 2023 and December 2023 were recruited as an independent external validation set. Univariate and multivariate binary logistic analyses were performed on the training set to select phenotypic variables to establish a nomogram. The discriminative performance of the model was evaluated using receiver operating characteristic (ROC) curves and calibration curves. Furthermore, the nomogram was validated in external validation sets.

**Results:**

Univariate and multivariate analyses identified independent trio-WES diagnosis-related predictive indicators, including severity of global developmental delay/intellectual disability, complexity of neurodevelopmental/neurological comorbid conditions, head circumference abnormalities, and brain malformations, in the training cohort and used to develop a nomogram. The nomogram showed excellent discrimination performance, with an area under curve (AUC) of the ROC in the training cohort of 0.868 (95% CI: 0.811–0.925), resulting in sensitivity, specificity, accuracy, precision, and F1 score values of 85.56, 82.05, 83.93, 84.62%, and 0.85, respectively. The model also exhibited strong prediction ability in the external validation set (AUC: 0.941, 95% CI: 0.880–0.998; sensitivity: 85.29%; specificity: 91.67%; accuracy: 87.93%; precision: 93.55%; and F1 score: 0.89). Moreover, the calibration curves demonstrated good agreement between the nomogram predictions and actual observations in both training and validation sets.

**Conclusion:**

We developed an user-friendly and highly accurate model for predicting the diagnostic probability of trio-WES in LF-ASDs children, which could help implement an individualized diagnostic strategy for affected children and their families at the pre-diagnosis stage.

## Introduction

Autism spectrum disorders (ASDs) represent a genetically and clinically heterogeneous group of neurodevelopmental disorders characterized by dysfunctions in social communication/interaction and repetitive, stereotypic patterns of movements/behaviors typically manifesting within the first 2–3 years of life (1). With advancements in understanding of ASDs, their prevalence has increased significantly, now accounting for approximately 1–2% of children worldwide, according to the Network Organization of Autism and Developmental Disabilities Monitoring (2). As of 2016, the prevalence of ASDs in children and adolescents (ages 3–17) in the United States was 2.76%, rising to 3.49% by 2020 (3, 4). Recently, the World Health Organization reported that 1 in 100 children worldwide present with ASDs (5). The rapidly increasing prevalence of ASDs places enormous pressure on public health systems, social services, and economic burdens on families worldwide.

Low-function ASDs (LF-ASDs) represent a severe manifestation within the ASDs continuum, affecting nearly 42% of diagnosed with ASDs children (2). LF-ASDs can be defined as ASDs accompanied by varying severities of global developmental delay or intellectual disability (GDD/ID), marked by pronounced and easily observable deficits from an early age (often <18 months) (6). Previous studies indicate that identifiable neurodevelopmental/neurological comorbid conditions (NCCs), such as attention deficit hyperactivity disorder (ADHD) and epilepsy (EP) frequently co-occur in children with LF-ASDs (1). Moreover, there is a stronger correlation with LF-ASDs and significant structural and functional brain alterations, including abnormalities in brain volume growth trajectories and pronounced cortical connectivity disturbances, which can complicate diagnosis and treatment; consequently, LF-ASDs are often referred to as syndromic ASDs. Therapeutic interventions or disease management for ASDs and LF-ASDs are individualized and multidisciplinary, typically encompassing speech and language therapy, occupational therapy, cognitive behavioral therapy, and pharmacotherapy (e.g., risperidone and aripiprazole).

The etiology of ASDs is complex and multifaceted, stemming from a combination of genetic predispositions and environmental influences. The etiology of LF-ASDs encompasses the foundational genetic and environmental factors associated with ASDs but is statistically associated with a higher burden of pathogenic genetic variants; thus, genetic disturbances are still considered to play essential roles in the development of LF-ASDs (1). For instance, mutations or chromosomal abnormalities with larger effect sizes, such as fragile X syndrome and single-gene disorders like Rett syndrome (*MECP2* abnormalities), are strongly associated with the etiology of LF-ASDs (ASDs with GDD/ID). Recent advancements in identifying the genetic components of LF-ASDs have accelerated, particularly due to the increased adoption and innovation of trio-based (parental-offspring model) whole-exome sequencing (trio-WES), the most common next-generation sequencing technology for detecting exon-level variants, including single-nucleotide variants (SNVs) and copy-number variants (CNVs) in clinical applications, making it possible to identify genetic components more frequently in many idiopathic LF-ASDs cases (7). Nonetheless, around half of children with LF-ASDs remain undiagnosed after receiving comprehensive trio-WES analyses, attributed to variants located outside exons (e.g., intronic, promoter, or enhancer-level variants); thus, the diagnostic yield of trio-WES for LF-ASDs remains unsatisfactory (1). Given the persistent challenges posed by the low diagnostic efficacy of trio-WES in LF-ASDs, it is imperative for pediatricians to develop practical tools for the early identification of children with LF-ASDs most likely be diagnosed by trio-WES, thereby facilitating timely evaluations of medical conditions. Additionally, due to the features of exon-level sequencing of trio-WES, it becomes essential for affected children and their families to use straightforward approaches to support their decision-making regarding the employment of trio-WES testing at the pre-diagnosis stage, ultimately aiding individualized family planning and reducing unnecessary financial and temporal costs.

Nomograms are powerful predictive tools, are widely used in forecasting the outcomes across various diseases due to their visualization, ease of use, objectivity, and accuracy (8). Nomograms have been widely used for predicting risks or outcomes of many pediatric neurodevelopmental disorders, including ADHD (9), infant neurodevelopmental delays (10), and teenager oppositional defiant disorder (11). However, to the best of our knowledge, no studies to date have reported the application of a nomogram for predicting the diagnostic efficacy of trio-WES in LF-ASDs children. Therefore, this multicenter study with independent external validation based on the phenotype-driven concept aims to generate the first user-friendly nomogram model for predicting the individualized diagnostic probability of trio-WES in children with LF-ASDs. Grounded in the phenotype-driven concept, which is essential in clinical genetics; this approach is used for Mendelian monogenic disorders to identify critical phenotypic characteristics that allow the identification of probands with a high probability of harboring relevant pathogenic genetic variants (12). Leveraging this concept, we used readily obtainable and objective phenotypic variables related to LF-ASDs and their associated complex NCCs, thereby establishing a predictive nomogram to assess the individualized diagnostic probability of employing trio-WES at the pre-diagnosis stage.

## Materials and methods

### Patients and subject selection criteria

**Figure 1:**
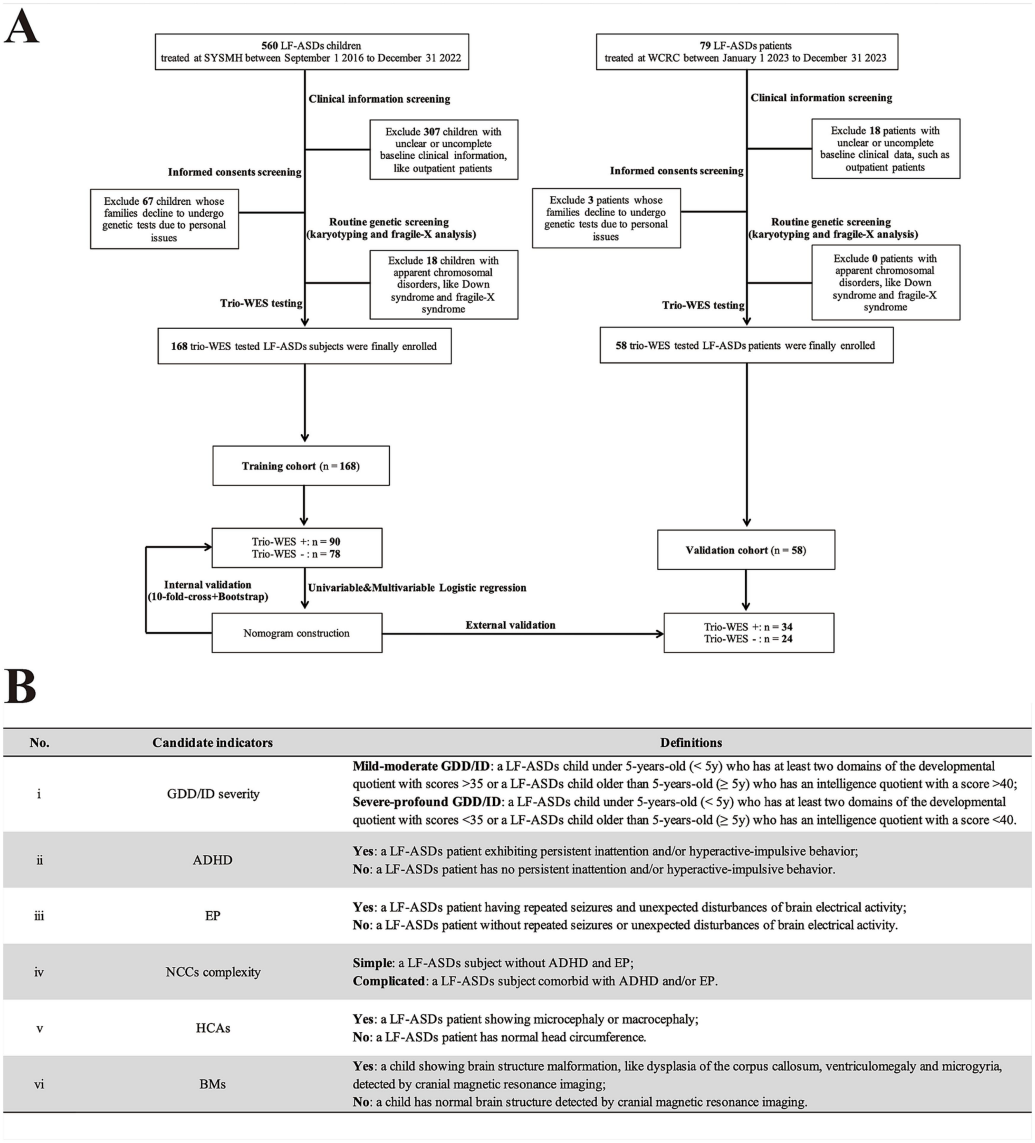
Flowchart and candidate variables description of this multicenter study. **(A)** Flowchart of this research. **(B)** A summary of candidate phenotypic indicators and their corresponding definitions in current research. LF-ASDs, low-function autism spectrum disorders; SYSMH, Sun Yat-Sen Memorial Hospital; WCRC, Weierkang Children’s Rehabilitation Center; trio-WES, trio-based whole-exome sequencing. NCCs, neurodevelopmental/neurological comorbid conditions; HCAs, head circumference abnormalities; BMs, brain malformations. In **A** “+/−” indicates enrolled LF-SADs patients who underwent trio-WES and received a positive genetic diagnosis (having “likely pathogenic” or “pathogenic” variants) or a negative genetic diagnosis (having “benign” or “uncertain significance” variants), respectively, according to the guidelines of the American College of Medical Genetics. In **B**, Gesell Developmental Diagnosis Scale with developmental quotient score (cut-off value: 35) was used to assess GDD severity (mild–moderate or severe-profound GDD) for patients under 5-year-old. While, Wechsler Intelligence Scale with intelligence quotient score (cut-off value: 40) was used to evaluate ID severity (mild–moderate or severe-profound ID) for patients older than 5-year-old.

As shown in Figures 1A, a total of 560 individuals diagnosed with LF-ASDs were admitted to the tertiary Children’s Medical Center of Sun Yat-sen Memorial Hospital (SYSMH) from September 2016 to December 2022. Following a comprehensive assessment involving clinical information, informed consent, and routine genetic screening (G-band karyotyping and fragile-X analysis) to exclude ineligible cases—such as children with unclear or incomplete clinical data (excluding 307 cases), those whose parents or guardians declined genetic testing or opted not to permit the use of their genetic results for publication (excluding 67 cases), and children presenting with apparent chromosomal disorders (e.g., Down syndrome or fragile-X syndrome), which rendered trio-WES inappropriate as a diagnostic strategy (excluding 18 cases), a total of 168 children with idiopathic LF-ASDs who had received trio-WES testing from SYSMH were ultimately in this retrospective study as training subjects. Moreover, 79 children diagnosed LF-ASDs were admitted to Weierkang Children’s Rehabilitation Center (WCRC), a specialized pediatric neurorehabilitation center focusing on integrated diagnosis and treatment of neurodevelopmental disorders, from January 2023 to December 2023. After performing a series of screenings similar to those applied in the SYSMH group, 58 patients with idiopathic LF-ASDs ultimately qualified for enrollment, having undergone trio-WES testing between January 1, 2023, and December 31, 2023, thus serving as external validation subjects.

The present study defined LF-ASDs in accordance with the following: (I) A clinical diagnosis of ASDs made by professional pediatric psychiatrists based on the Diagnostic and Statistical Manual of Mental Disorders, 5th Edition (DSM-5) criteria for ASDs (13), supplemented by several main ASDs-related clinical assessment scales, including the Modified Checklist for Autism in Toddlers, the Clancy Autism Behavior Scale, the Autism Behavior Checklist, and the Childhood Autism Rating Scale (14–16). (II) Diverse severities of GDD/ID, where by clinical diagnostic criteria for GDD/ID were based on the DSM-5 criteria (13). GDD/ID severity was assessed mainly according to the Gesell Developmental Diagnosis Scale (GDDS) for infants under 3 year-old (17), and Wechsler Preschool and Primary Scale of Intelligence, IV Edition (WPPSI-IV) for children with 4 ~ 6 year-old. For subjects over 6 year-old, we used Wechsler Intelligence Scale for Children, IV Edition (WISC-IV) to assess their GDD/ID severity (18). (III) Exclusion of subjects with identified non-genetic causes such as hypoxic–ischemic encephalopathy, bilirubin encephalopathy and intrauterine infections, and positive findings from routine genetic screens (fragile-X analysis and G-band karyotyping) that indicate chromosomal disorders, such as fragile X syndrome or Down syndrome, deemed inappropriate for trio-WES in those conditions. (IV) Children with or without common NCCs, mainly ADHD and/or epilepsy, with diagnoses established by specialized child psychiatrists and neurologists following the criteria of the DSM-5 for ADHD (13) and the International League Against Epilepsy (ILAE) criteria for epilepsy (19). Due to the diagnostic challenges and lack of objective and Chinese version of assessment tools for other NCCs such as sleep disorder and anxiety disorder in young children, these conditions were excluded from current study.

### Ethical compliance

The design of this multicenter study received approval from the Ethical Committee of Sun Yat-sen Memorial Hospital, Sun Yat-sen University (initiative affiliation approval number: SYSKY-2025-244-01). Written informed consent for genetic investigation and publication of genetic results was obtained from the parents or guardians of all 226 enrolled subjects.

### Strategy for variant capture of trio-WES

The principles of the variant capture process and quality control systems for trio-WES have been described in previous studies (20–22), with details in the current study briefly outlined as follows: Genomic DNA was extracted from the whole blood of the proband and their parents using a commercial genomic extraction kit (Qiagen, Shanghai, China). The Illumina TruSeq Exome Kit (Illumina, San Diego, CA, United States) was used for DNA library construction and the generation of approximately 10GB of exome sequencing data per individual. GeneRanger (Xunyin Biotech, Shanghai, China) was employed for the exome sequencing data analysis. Subsequently, the Burrows-Wheeler aligner, Picard tool, and genome analysis tools were employed for read alignment, indel region realignment, base quality recalibration, variant capture, and calling/transformation based on the Genome Aggregation Database (gnomAD). The variant quality control system was set to a coverage depth of greater than 10, along with a minor allele frequency of less than 0.05%.

### Pathogenicity criteria for trio-WES-identified exon-level variants and enrolled case grouping

The pathogenicity of trio-WES-identified SNVs was rated according to the 2015 American College of Medical Genetics (ACMG) guidelines for SNV interpretation (23), categorizing detected SNVs into “pathogenic/likely pathogenic” and “benign/uncertain significance” SNVs. As per prior research (24), gnomAD and in-house SNV population frequency databases were used to assess SNV allele frequency. *In silico* pathogenic predictions for identified missense, frameshift, nonsense, and deletion variants were conducted using online versions of Mutation Taster,^1^ Protein Variation Effect Analyzer,^2^ Polymorphism Phenotyping version 2,^3^ and Sorting Intolerant From Tolerant.^4^ *In silico* prediction for the splice variant was conducted using online version of Combined Annotation Dependent Depletion.^5^ Additionally, the Human Genomic Mutation Database and PubMed were consulted to determine whether identified variants had been previously documented, while Online Mendelian Inheritance in Man^6^ database was employed to obtain genotype–phenotype profiles linked to identified SNVs.

The pathogenicity of trio-WES-detected CNVs was assessed based on the 2019 ACMG guidelines for postnatal CNV interpretation (25), employing previously documented methods (26). Identified CNVs were manually interpreted and categorized into “pathogenic/likely pathogenic” or “benign/uncertain significance” by two or more experienced clinical geneticists adhering to ACMG guidelines.

Based on the pathogenicity assessments of these trio-WES-identified SNVs and CNVs mentioned above, enrolled subjects were categorized into LF-ASDs children with positive genetic diagnoses (+, harboring pathogenic/likely pathogenic SNVs or CNVs from their trio-WES testing reports) and LF-ASDs children with negative genetic diagnoses (−, harboring benign/uncertain significance SNVs or CNVs from their trio-WES testing reports).

### Candidate variable collection and interpretation of collected indicators

Demographic and phenotypic factors of all enrolled subjects were collected from hospital medical records. These included: (I) demographic characteristics including sex, admission date, and age at which trio-WES was performed; and (II) candidate phenotypic factors: GDD/ID severity, ADHD, epilepsy, complexity of NCCs, head circumference abnormalities (HCAs), and brain malformations (BMs). A summary table detailing these phenotypic variables and their corresponding definitions, is provided in Figure 1B.

### Model development and internal/external validation of model performance

For model development, independent phenotypic indicators were first screened through univariate and multivariate binary logistic regression alongside collinearity diagnostic analyses in the training set. Specifically, during the univariate and multivariate logistic analyses of the training set, indicators with clinical significance and significantly significant differences (*p* < 0.05) were identified between children with positive and negative genetic diagnoses via trio-WES. Then, collinearity diagnostic analyses were performed to determine the presence of significant collinearity among the screened indicators. Tolerance and variance inflation factor (VIF) metrics were used to evaluate the severity of collinearity. A tolerance value exceeding 0.5 and a VIF below 5 for each screened variable indicated no significant collinearity, thereby permitting the selection of these variables as independent for establishing the logistic regression model (27). Finally, we generated a nomogram using R packages to visualize the constructed logistic regression model.

For internal validation of model performance, the receiver operating characteristic (ROC) curve and the area under curve (AUC) of the ROC were initially used to evaluate the discriminative performance of the model in the training cohort. Subsequently, calibration curves coupled with the Hosmer-Lemeshow test were applied to assess the goodness-of-fit between predicted and observed data. A *p* value from the Hosmer-Lemeshow test <0.05 indicated that the dotted line (representing model-predicted data) significantly differed from the solid line (representing actual observed data) in calibration curve, demonstrating poor model fit; conversely, a *p* value >0.05 implied good model fit. Additionally, the clinical applicability of the nomogram was evaluated through decision curve analysis (DCA) and clinical impact curve (CIC). Furthermore, we used two methods to assess consistency and mitigate overfitting bias: the 10-fold cross-validation and bootstrap resampling (with 1,000 bootstrap resamples). The 10-fold cross-validation approach is a common and robust resampling technique used to assess the consistence performance and internal stability of predictive model, and involves partitioning the dataset into 10 mutually exclusive and approximately equal-sized folds. During iterative training, 9 folds are used as the training set while the remaining fold serves as the validation set. This process is repeated across all folds. The established performance metric, concordance index (C-index), is calculated to examine the consistency (generalization) and stability of predictive model. Conversely, the bootstrap sampling method, a classical internal validation method, draws from the original training dataset with replacement and undergoes 1,000 repetitions. C-index values greater than 0.7 from both methods indicated the nomogram had good reliability (28).

For external validation of model performance, the optimal cutoff value was first set based on the maximal Youden index value corresponding to the optimal values of sensitivity and specificity of the model in the training set. Cases in the external validation set were then classified into “nomogram-predicted positive diagnostic cases” and “nomogram-predicted negative diagnostic cases” based on these optimal cutoff values. The AUC value, calibration curves with the Hosmer-Lemeshow test, and DCA/CIC, were subsequently used to validate the discriminative performance, consistency, and clinical benefits of the nomogram in the external validation set. Finally, we calculated the model sensitivity, specificity, accuracy, precision, and F1 scores for the training and external validation sets, and the results were visualized using Sankey plots.

### Statistical analysis

Microsoft Excel software was used for data entry, while all statistical analyses were conducted using R.^7^ As referenced in previous studies using the R (8, 27, 29, 30), the following R packages were used for statistical analysis and data visualization: ggplot2,” “foreign,” “rms,” “rmda,” “caret,” “tidyverse,” and “ggDCA.” *p* value <0.05 were considered statistically significant.

## Results

### Clinical details of the enrolled subjects

In total, 168 and 58 children with unexplained LF-ASDs were enrolled in the training and external validation cohorts, respectively. Comparisons of the baseline demographics and phenotypic features between the two cohorts are shown in Table 1. Detailed data regarding the genotypes and phenotypes of subjects in the training and validation cohorts are summarized in Supplementary Files 1–4, respectively. Among the 168 enrolled subjects in the training cohort, 90 (53.6%) had a genetic diagnosis via trio-WES, whereas 78 (46.4%) did not have a genetic diagnosis via trio-WES. Moreover, 58.6% (34/58) of the individuals included in the external validation cohort received a genetic diagnosis via trio-WES.

**Table 1:** Comparison of baseline demographics and phenotypic features between training and validation cohorts of trio-WES tested LF-ASDs children.

Demographics or characteristics	Indicators	Training cohort	Validation cohort	*t/χ2* value	*p*-value
Demographic parameters					
Case number (*n*)		168	58		
Sex [*n* (%)]	Female	43 (25.6%)	16 (27.6%)	0.089	0.766
	Male	125 (74.4%)	42 (72.4%)		
Admission Periods (MM/YY ~ MM/YY)		Sep/2016 ~ Dec/2022	Jan/2023 ~ Dec/2023		
Age of receiving trio-WES [Mean ± SD/(yrs)]		4.7 ± 3.4	5.3 ± 2.6	1.324	0.188
Patient sources		SYSMH	WCRC		
Phenotypic features					
GDD/ID severity [*n* (%)]	Mild–moderate	92 (54.8%)	28 (48.3%)	0.728	0.393
	Severe-profound	76 (45.2%)	30 (51.7%)		
ADHD [*n* (%)]	Yes	34 (20.2%)	18 (31.0%)	2.837	0.092
	No	134 (79.8%)	40 (69.0%)		
EP [*n* (%)]	Yes	27 (16.1%)	11 (19.0%)	0.258	0.611
	No	141 (83.9%)	47 (81.0%)		
NCCs complexity [*n* (%)]	Simple	124 (73.8%)	30 (51.7%)	9.687	0.002
	Complicated	44 (26.2%)	28 (48.3%)	0.065	0.798
HCAs [*n* (%)]	Yes	52 (31.0%)	19 (32.8%)		
	No	116 (69.0%)	39 (67.2%)		
BMs [*n* (%)]	Yes	70 (41.7%)	15 (25.9%)	4.590	0.032
	No	98 (58.3%)	43 (74.1%)		
Outcomes					
trio-WES-based diagnostic status [*n* (%)]	Positive	90 (53.6%)	34 (58.6%)	0.444	0.505
	Negative	78 (46.4%)	24 (41.4%)		

LF-ASDs, low-function autism spectrum disorders; NCCs, neurodevelopmental/neurological comorbid conditions; trio-WES, trio-based whole exome sequencing SYSMH, Sun Yat-Sen Memorial Hospital; WCRC, Weierkang Children’s Rehabilitation Center; GDD/ID, global developmental delay/intellectual disability; ADHD, attention defi cit hyperactivity disorder; EP, epilepsy; HCAs, head circumference abnormalities; BMs, brain malformations. In NCCs complexity row, “simple” means patients only existing LF-ASDs without ADHD and (or) EP; “complicated” means LF-ASDs patients coexisting one or two type of other NCCs (ADHD and EP).

### Independent predictive variable screening and logistic regression model establishment

As shown in Table 2, the univariate logistic analysis revealed that five phenotypic indicators (GDD/ID severity, ADHD, NCC complexity, HCAs, and BMs) were potentially associated with a positive trio-WES diagnosis.

**Table 2:** Univariate and multivariate logistic regression for predicting diagnostic efficacy of using trio-WES in 168 LF-ASDs children in training cohort.

Univariate logistic analysis			Multivariate logistic analysis		
Candidate indicators	OR (95% CI)	*p* value	Candidate indicators	OR (95% CI)	*p* value
Age of receiving trio-WES	0.981 (0.898–1.072)	0.675			
GDD/ID severity	15.836 (7.209–34.788)	< 0.001 ***	GDD/ID severity	11.264 (4.788–26.495)	< 0.001 ***
ADHD	2.473 (1.098–5.568)	0.029 *			
EP	1.584 (0.678–3.698)	0.288			
NCCs complexity	3.034 (1.431–6.433)	0.004 **	NCCs complexity	2.671 (1.055–6.764)	0.038 *
HCAs	5.950 (2.721–13.011)	< 0.001 ***	HCAs	2.801 (1.070–7.332)	0.036 *
BMs	5.140 (2.599–10.167)	< 0.001 ***	BMs	3.701 (1.601–8.558)	0.002 **

trio-WES, trio-based whole exome sequencing; LF-ASDs, low-function autism spectrum disorders; GDD/ID, global developmental delay/intellectual disability; NCCs, neurodevelopmental/neurological comorbid conditions; ADHD, attention deficit hyperactivity disorder; EP, epilepsy; HCAs, head circumference abnormalities; BMs, brain malformations; OR (95%CI), odds ratio (95% confidence interval). *p < 0.05; **p < 0.01; ***p < 0.001.

Following the univariate logistic analysis, the five candidate indicators were incorporated into a multivariate logistic regression model. As shown in Table 2, the multivariate analysis results indicated that GDD/ID severity (OR: 11.264; 95% CI: 4.788–26.495, *p* < 0.001), NCC complexity (2.671; 1.055–6.764, *p* < 0.05), HCAs (2.801; 1.070–7.332, *p* < 0.05), and BMs (3.701; 1.601–8.558, *p* < 0.01) were independently associated with a higher diagnostic efficacy when applying trio-WES in patients with LF-ASDs. In contrast, having ADHD was not independently associated with a higher possibility of receiving a genetic diagnosis by trio-WES in LF-ASDs children.

Collinearity diagnosis performed on the four candidate indicators (GDD/ID severity, NCC complexity, HCAs, and BMs) showed no significant evidence of collinearity, as the tolerances and the VIFs for each phenotypic factor were all >0.5 and <5, respectively (Table 3).

**Table 3:** The collinearity diagnostic analysis of indicators for predicting diagnostic efficacy of using trio-WES in LF-ASDs children in training cohort.

Candidate variables	Tolerance	VIF
GDD/ID severity	0.822	1.216
NCCs complexity	0.967	1.034
HCAs	0.823	1.215
BMs	0.891	1.123

Trio-WES, trio-based whole exome sequencing; LF-ASDs, low-function autism spectrum disorder; GDD/ID, global developmental delay/intellectual disability; ADHD, attention deficit hyperactivity disorder; NCCs, neurodevelopmental/neurological comorbid conditions; HCAs, head circumference abnormalities; BMs, brain malformations; VIF, variance inflation factor.

Finally, based on the four-variable binary logistic regression *β* values and the intercept term, a regression model was established to predict the diagnostic efficacy of applying trio-WES in children with LF-ASDs. The corresponding formula for predicting the probability (P) of an individual with LF-ASDs being diagnosed by trio-WES is as follows: Logit (P) = 2.422 (*β*_1_) × GDD/ID severity (severe-profound: 1; mild–moderate: 0) + 0.983 (β_2_) × NCCs complexity (complicated: 1; simple: 0) + 1.030 (β_3_) × HCAs (yes: 1; no: 0) + 1.309 (*β*_4_) × BMs (yes: 1; no: 0) – 1.898 (intercept term).

### Predictive model visualization and nomogram usage

**Figure 2:**
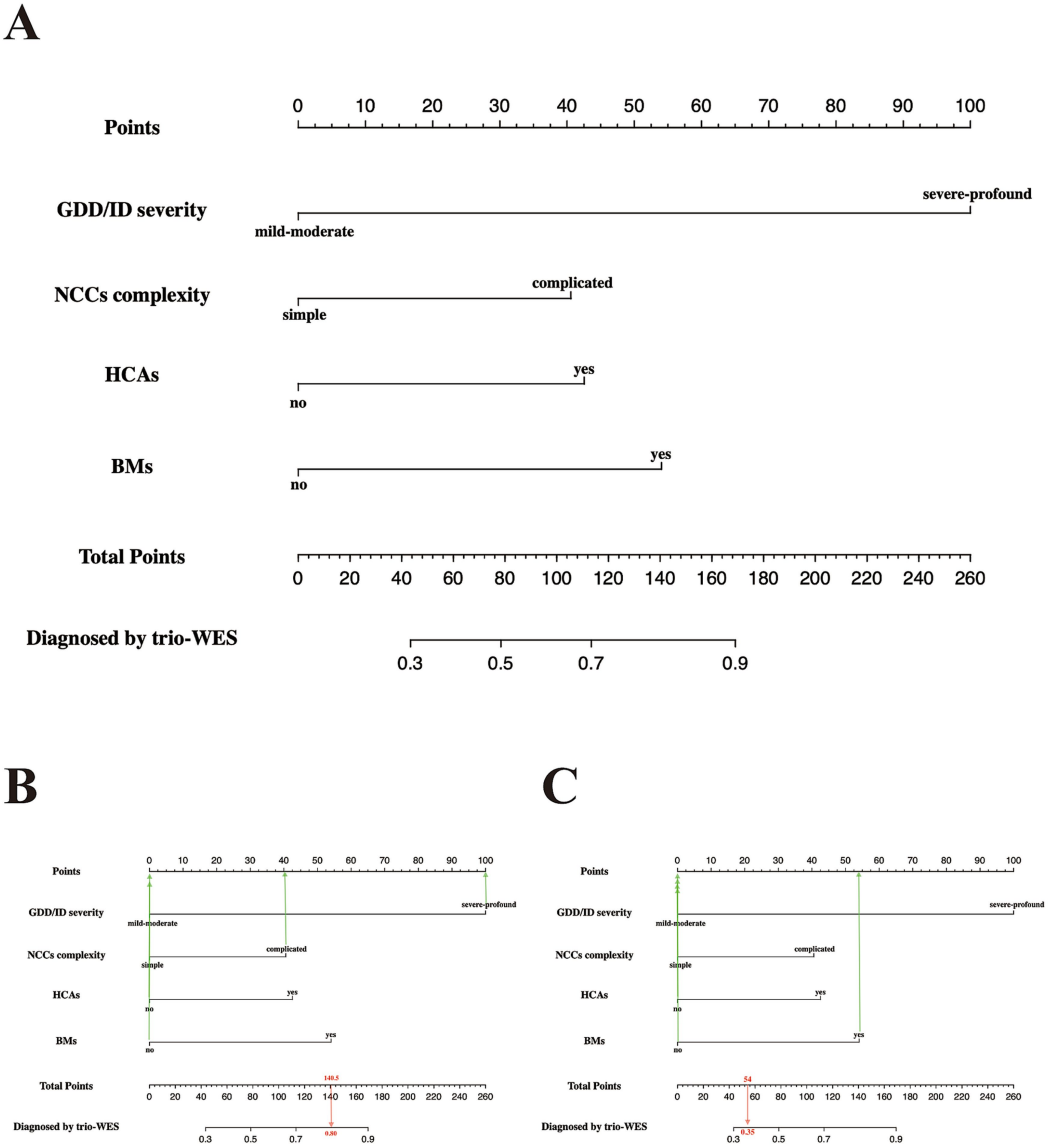
Predictive nomogram for LF-ASDs children in the training set, estimating the probability of receiving a positive genetic diagnosis by applying trio-WES. **(A)** The nomogram plot has two parts: the top portion (from the “Point” section to the last the “BMs” section) is designed to calculate the respective point scores of each incorporated phenotypic indicator. The bottom portion (from the “Total Points” section to the “Diagnosis by trio-WES” section) is used to analyze the probability of having a positive genetic diagnosis via trio-WES for each enrolled LF-ASDs subject. **(B,C)** represent two examples with high and low probabilities of receiving a genetic diagnosis via trio-WES, respectively. The red arrow in **(B)** reveals an LF-ASDs child with an approximate total score of 154, and the matched predicted probability of receiving a genetic diagnosis via trio-WES is approximately 85%, whereas the red arrow in **(C)** indicates an LF-ASDs subject with an approximate total score of 54. The matched predicted probability of receiving a genetic diagnosis via trio-WES is approximately 35%. LF-ASDs, low-function autism spectrum disorders; GDD/ID, global developmental delay/intellectual disability; NCCs, neurodevelopmental/neurological comorbid conditions; HCAs, head circumference abnormalities; BMs, brain malformations; trio-WES, trio-based whole-exome sequencing.

Figure 2 shows the nomogram plot based on the established logistic regression model. This nomogram provides an estimate of the individual probability of diagnosis via trio-WES through a score-contribution system and is designed for a child with LF-ASDs at the time of initial admission or during the pre-diagnosis stage. The methodology for this score-contribution system utilizes the coefficients (β) from the logistic regression model. The calculated score assigned to each indicator is proportional to its β value, mapped to a 0 to 100-point scale through linear transformation. Specifically, we assigned a value of 100 to the indicator with the maximum β value (β_max_), from which the scores for the other predictive indicators can be calculated using the formula: calculated score_x_ = 100 × β_x_ ÷ β_max_. Detailed parameters related to this regression model and the corresponding model scores for each independent predictive variable are provided in Table 4.

**Table 4:** Coefficients of binary logistic regression for predicting diagnostic efficacy via trio-WES in individuals with LF-ASDs in training set.

Phenotypic variables	B	S. E.	Wald	*p* value	OR	95% CI for OR	Calculated score
GDD/ID severity	2.422 (β1)	0.436	30.789	<0.001	11.264	4.788–26.495	100*
NCCs complexity	0.983 (β2)	0.474	4.297	0.038	2.671	1.055–6.764	40.5 (100 × β2÷*β*1)
HCAs	1.030 (β3)	0.491	4.402	0.036	2.801	1.070–7.332	42.5 (100 × β3÷β1)
BMs	1.309 (β4)	0.428	9.364	0.002	3.701	1.601–8.558	54 (100 × β4÷β1)

Trio-WES, trio-based whole exome sequencing; LF-ASDs, low-function autism spectrum disorders; GDD/ID, global developmental delay/intellectual disability; NCCs, neurodeveopmental/neurological comorbid conditions; HCAs, head circumference abnormalities; BMs, brain malformations; B, β value; S. E., standard error; OR, odds ratio; 95% CI, 95% confidence interval. * The variable showing the max β value and being set 100-point as reference-point for other included variables.

As indicated in Figure 2A, LF-ASDs with severe-profound GDD/ID had the greatest influence on the probability of receiving a diagnosis through trio-WES, followed by BMs, HCAs, and complicated NCCs (NCC with coexisting ADHD and/or epilepsy). For example, a 4.5-year-old girl with LF-ASDs (subject no. 89 in the training group) with severe-profound GDD and comorbid ADHD and epilepsy (complicated NCCs), but without BMs and HCAs, achieved a total score of approximately 140.5 points; the corresponding probability of obtaining a genetic diagnosis through trio-WES was approximately 80%. Indeed, this girl was diagnosed with “Developmental and Epileptic Encephalopathy 2 (OMIM#300672)” through trio-WES (Figure 2B). Another subject with LF-ASDs, a 9-year-old boy (subject No. 84 in the training cohort) with mild–moderate ID and multiple BMs detected via brain MRI, including basal ganglia lesions, pituitary dysplasia, and cerebellar atrophy, but without HCAs, ADHD, and epilepsy (simple NCCs), scored approximately 54 points, with the corresponding probability of a positive genetic diagnosis via trio-WES being approximately 35%. This boy has yet to receive a genetic diagnosis despite his multiple brain anomalies following comprehensive trio-WES analysis and re-evaluation of the trio-WES data (Figure 2C).

### Assessment and internal validation of the nomogram performance in the training set

**Figure 3:**
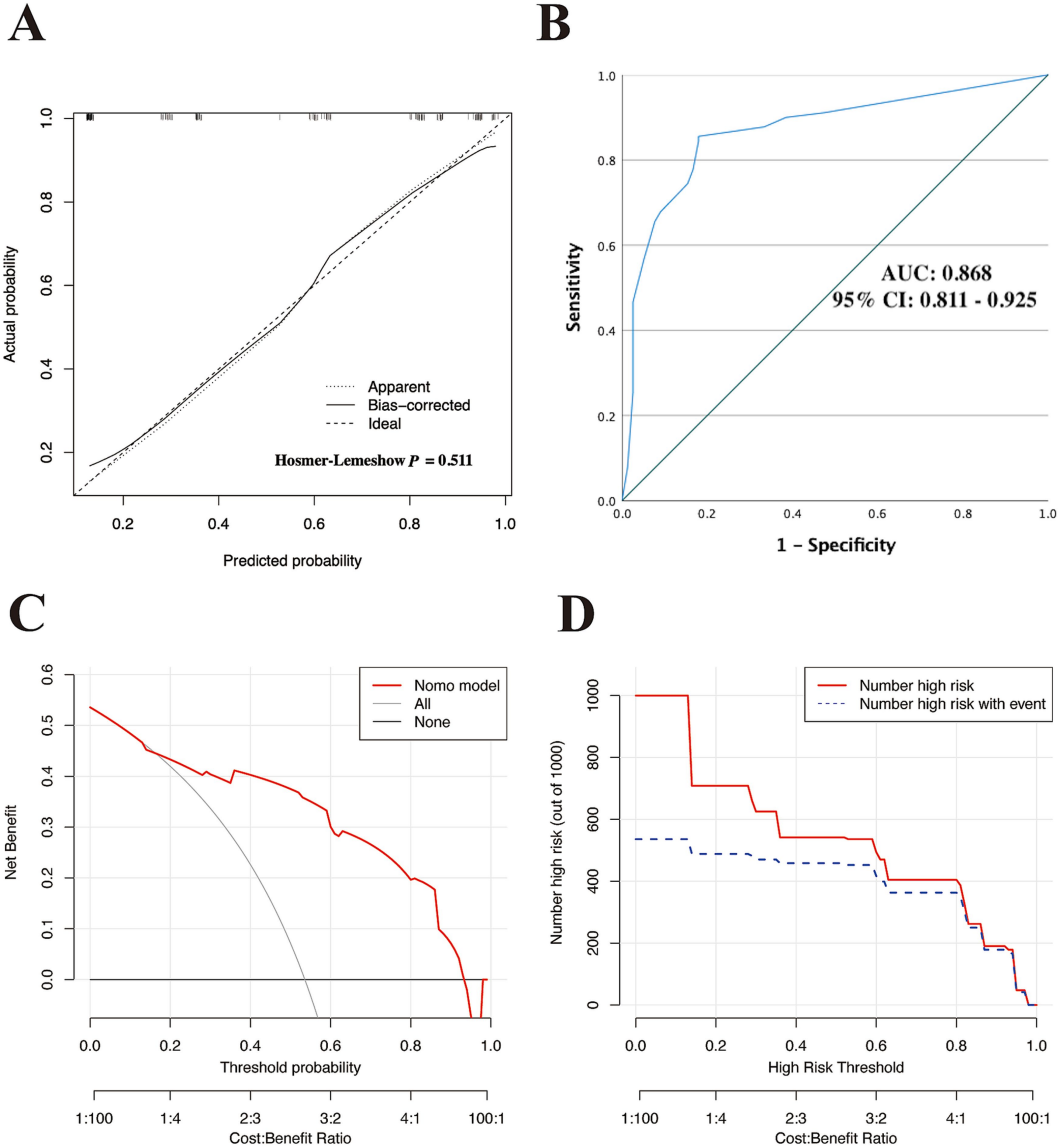
Assessment of the discriminatory performance of the nomogram in the training cohort. **(A)** Calibration plot with Hosmer–Lemeshow test. **(B)** ROC curve for evaluating the nomogram-predicted accuracy in the training set. DCA **(C)** and CIC **(D)** were used to determine the predicted clinical utility and clinical impact of the model in the training cohort. ROC, receiver operating characteristic; AUC, area under curve of the ROC; 95% CI, 95% confidence interval; DCA, decision curve analysis; CIC, clinical impact curve.

First, a calibration curve with Hosmer-Lemeshow testing was used to evaluate the fitness of the nomogram model within the training cohort. As demonstrated in Figure 3A, the calibration analysis indicated a good fit between the observed and model-predicted diagnostic probabilities (*χ*^2^ = 4.275; *p* value = 0.511), indicating satisfactory consistency between the predicted and observed values.

**Figure 4:**
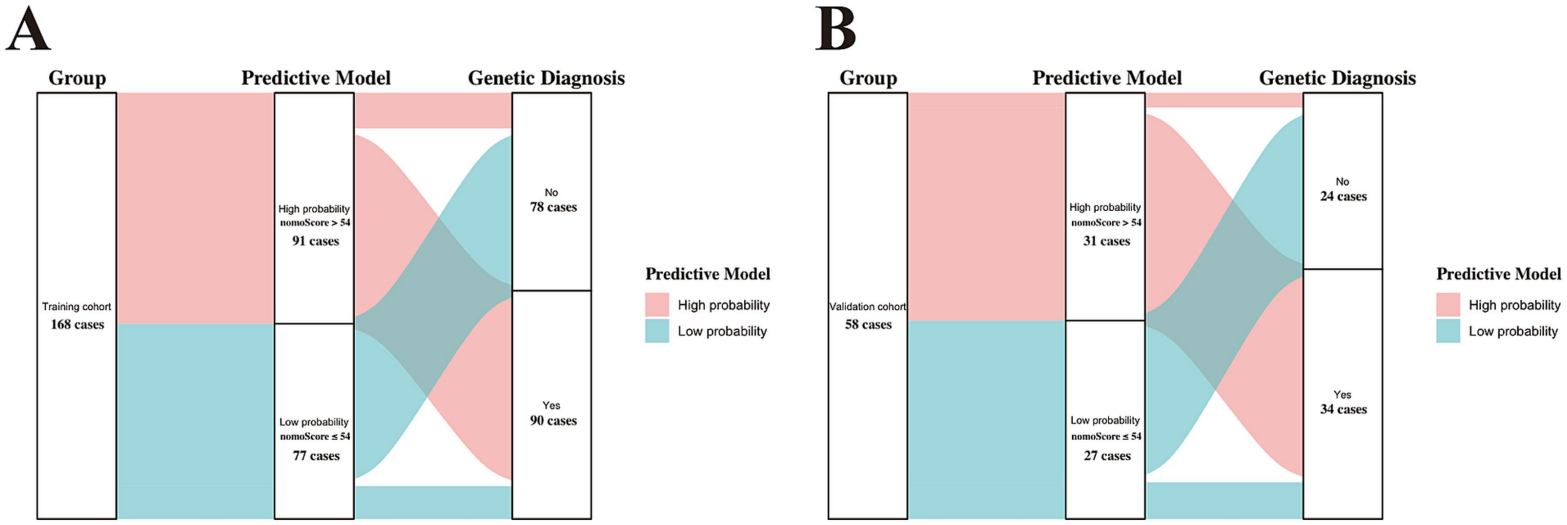
Sankey plots showing the discriminatory performance of the predictive model in the training **(A)** and validation **(B)** cohorts. nomoScore, nomogram score. the calculated maximal Youden index (0.677) based on the training set was selected to set the optimal cutoff value of the nomoScore (54), a critical value that clustered the two groups (training and validation cohorts) into subgroups with high and low probabilities of receiving positive genetic diagnosis by trio-WES.

Subsequently, we used ROC curves to evaluate the discriminative ability of the nomogram. Figure 3B demonstrates an AUC of 0.868 (95% CI: 0.811–0.925), indicating good predictive performance in the training cohort. Based on the ROC plot of the training set, the maximal Youden index was 0.677 and was used to establish the optimal cutoff nomogram score (nomoScore) value = 54, generating a confusion matrix that yielded sensitivity, specificity, accuracy, precision, recall, and F1 score values of 85.56, 82.05, 83.93, 84.62, 85.56%, and 0.85, respectively, in the training set (Figure 4A; Table 5). These findings further underscore the nomogram’s promising capability in predicting the diagnostic probability of applying trio-WES in the diagnostic strategy of LF-ASDs in children.

**Table 5:** Predictive performance of the constructed phenotype-driven nomogram model in training and validation sets.

Predictive values	Training set	Validation set
Sensitivity (%)	85.56%	85.29%
Specificity (%)	82.05%	91.67%
Accuracy (%)	83.93%	87.93%
Precision (%)	84.62%	93.55%
Recall (%)	85.56%	85.29%
F1 score	0.85	0.89

Additionally, we used DCA and the CIC to assess the clinical usefulness of the nomogram model in the training set. As demonstrated in Figures 3C–D, children with LF-ASDs could receive greater net benefits from this nomogram compared to hypothetical treat-none or treat-all scenarios, suggesting that applying this model to predict the diagnostic efficacy of trio-WES for LF-ASDs patients may yield significant benefits.

**Figure 5:**
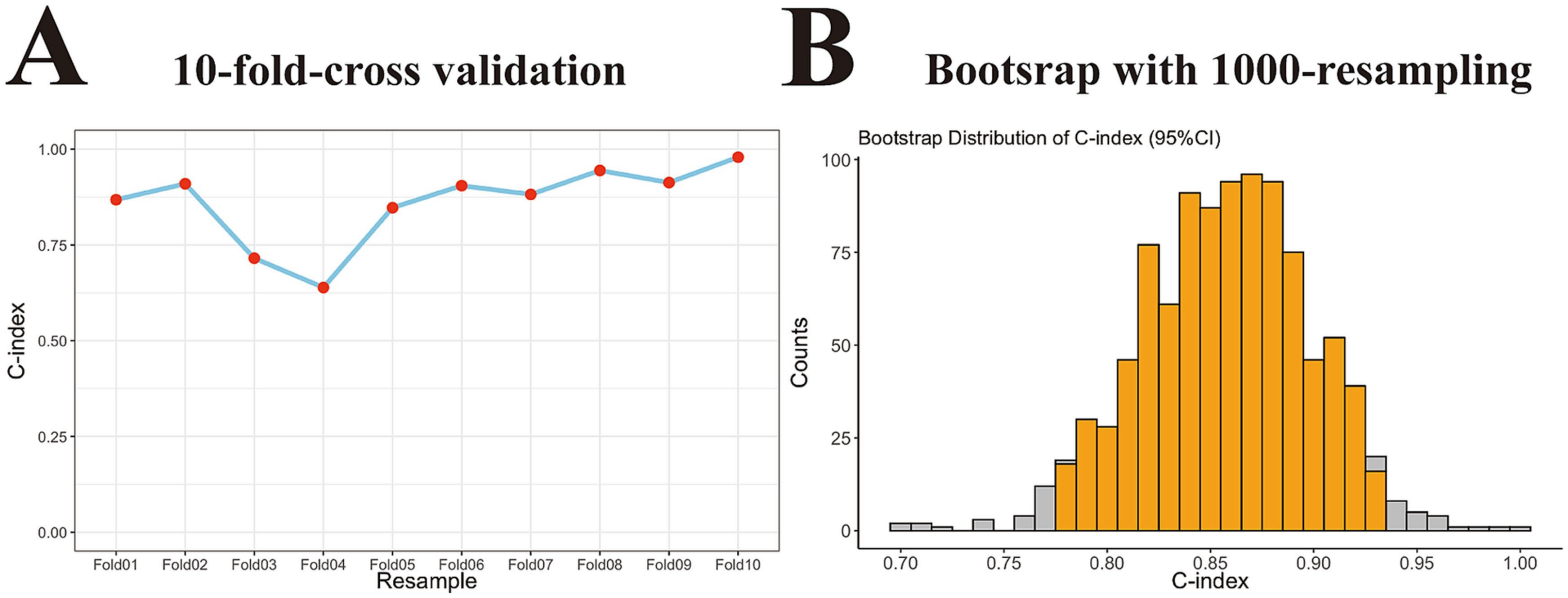
Internal validation of the generalization performance of the nomogram model in training cohort. **(A)** Point-fold line chart with 10-fold-cross validation approach showing the nomogram had good stability with excellent consistence in training set (C-index, 0.860 with 95% CI, 0.785–0.935). **(B)** Histogram with 1,000-time resampling bootstrap method revealing the nomogram did not overfit in training set (C-index, 0.856 with 95% CI, 0.776–0.939). C-index, concordance index; 95% CI, 95% confidence interval.

Finally, we used 10-fold cross-validation and bootstrapping re- for internal validation to determine the generalization performance of the nomogram. As depicted in Figure 5A C-index value following the 10-fold cross-validation was 0.860 (95% CI: 0.785–0.935). Similarly, the bootstrap method with 1,000 resamples yielded a C-index of 0.856 (95% CI: 0.776–0.939; Figure 5B). Collectively, these results indicate that the nomogram exhibits good stability with excellent consistence and no evidence of overfitting.

### External validation of the nomogram performance in the independent validation set

**Figure 6:**
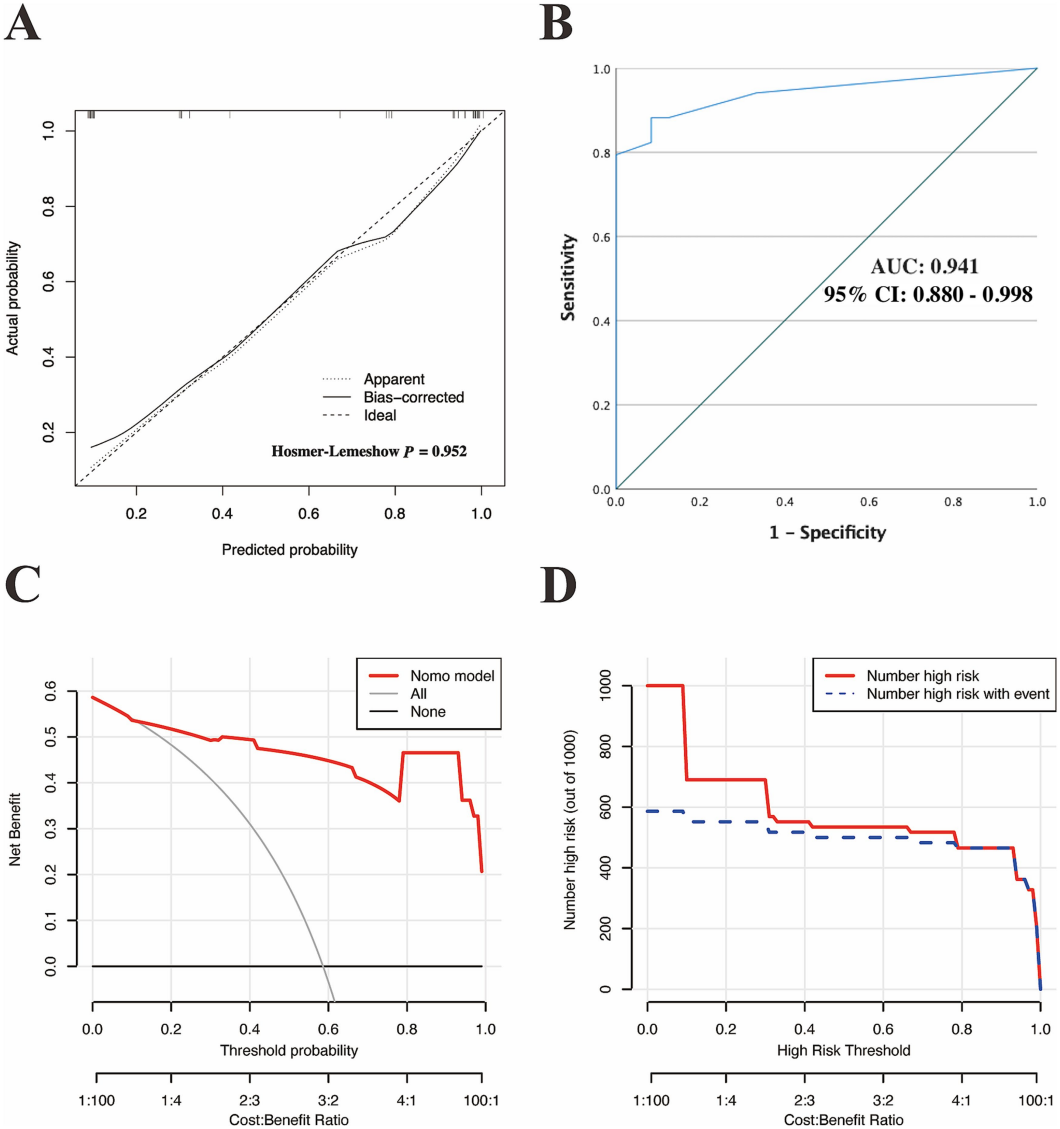
External validation of the discriminatory performance of the nomogram model in an independent external cohort. **(A)** Calibration plot with Hosmer–Lemeshow test. **(B)** ROC curve verifying the nomogram-predicted accuracy in the external set. DCA **(C)** and the CIC **(D)** were used to verify the clinical value of the model in an external cohort. ROC, receiver operating characteristic; AUC, area under curve of the ROC; 95% CI, 95% confidence interval; DCA, decision curve analysis; CIC, clinical impact curve.

Based on the optimal cutoff value (nomoScore = 54), all 58 cases within the independent validation cohort were classified into 31 nomogram-predicted positive diagnostic cases and 27 nomogram-predicted negative diagnostic cases. As illustrated in Figure 6A, the calibration curve with the Hosmer-Lemeshow test demonstrated excellent agreement between the nomogram-predicted values and the actual observed results (*χ*^2^ = 1.125, *p* value = 0.952) in the transformed external set. Additionally, a ROC plot was used to validate the discriminative performance of the model in the transformed external set, which revealed robust discriminative ability (AUC: 0.941; 95% CI: 0.880–0.998; Figure 6B). The results of DCA and CIC within the transformed external cohort indicated that employing this nomogram to predict the diagnostic efficacy of trio-WES for LF-ASDs children could yield significant net benefits (Figures 6C,D). The confusion matrix results illustrated in the Sankey plot for the external set revealed sensitivity, specificity, accuracy, precision, and F1 score of the nomogram were 85.29, 91.67, 87.93, 93.55%, and 0.89, respectively (Figure 4B; Table 5). These external validation results suggest that the proposed model demonstrates stable reproducibility and robust repeatability.

## Discussion

The rapid development of next-generation sequencing technology has enabled the identification of genetic components in many children with unexplained neurological syndromes, including LF-ASDs (24, 31–33). The application of trio-WES has transformed the landscape of clinical genetics, facilitating a more cost-effective means of obtaining diagnoses for various Mendelian disorders compared to traditional genetic tests, such as target-panel sequencing with family phenotype segregation analysis. This shift has alleviated the “diagnostic odysseys” frequently encountered by affected children and their families (34–37). However, it is essential to recognize the technical limitations of trio-WES and the complexities associated with human genomic disturbances, such as intronic structural variants and non-coding variants, which may hinder its diagnostic effectiveness. Whole-genome sequencing (WGS) could potentially address these limitations by facilitating the identification of intronic structural or non-coding variants. Nevertheless, the high costs associated with WGS significantly restrict its clinical application and widespread use as a routine genetic diagnostic approach is largely restricted (38, 39). To date, trio-WES remains the first-tier genetic diagnosis option globally and constitutes a critical element of subsequent genetic counseling and patient management for many Mendelian disorders (40). Thus, developing effective approaches and tools to analyze the diagnostic rate of trio-WES in clinical contexts related to various idiopathic and complex disorders, including LF-ASDs, remains a pertinent endeavor.

Numerous factors, such as disorder type, disease onset age, and variant capture strategy, may influence the diagnostic efficacy of trio-WES in clinical practice (12, 38). For instance, the diagnostic yield of trio-WES may reach 92% in children with idiopathic dermatological syndromes (12), potentially due to the clear presentation and delineation of phenotypic information evident in those conditions, which likely contributes to the elevated diagnostic rate achievable through trio-WES. Thus, accurate and comprehensive assessments of clinical phenotypes at the pre-diagnosis stage are paramount and pose considerable challenges, necessitating rigorous collection and precise analysis of various phenotypic features for every patient. In this study, we meticulously recorded all associated phenotypic clues that accurately reflect the neurological conditions of each child with LF-ASDs to enhance the diagnostic yield. The global diagnostic yields in the training and external validation groups were 53.6 and 58.6%, respectively; these figures are similar to those documented in a previous WES report involving LF-ASDs children with additional associated conditions (51.3%) (1). Our findings regarding the global diagnostic rate associated with the implementation of trio-WES in children with LF-ASDs further reinforce prior conclusions that complex phenotypic features—encompassing multiple neurological disorders (including ADHD and/or epilepsy), multiple neurological anomalies (such as HCAs and/or BMs), and severe-profound levels of cognitive or developmental impairment—are more likely linked to an exon-level variant within the clinical setting. This observation implies a potential relationship between the enrichment of phenotypic characteristics and the diagnostic yield of trio-WES in children with LF-ASDs (1, 24). We thus propose the possibility of establishing a diagnostic predictive model for the application of trio-WES in LF-ASDs patients by incorporating key phenotypic factors associated with a higher probability of obtaining genetic results. This model could help pediatricians make more appropriate and personalized management for affected children during the pre-diagnosis stage.

The present study identified four key phenotypic indicators (GDD/ID severity, NCC complexity, HCAs, and BMs) as being associated with the possibility of obtaining genetic results through trio-WES in children with LF-ASDs. We hypothesize that severe-profound GDD/ID, BMs, HCAs, and a broad spectrum of NCCs associated with LF-ASDs may share common genetic backgrounds linked to overlapping genetic factors, which ultimately results in a higher trio-WES diagnostic rate. Over 1,200 genes related to ASDs susceptibility (called ASDs-related genes) have been cataloged in the Simons Foundation Autism Research Initiative^8^ gene dataset (41); the two major gene fall into two functional categories: those involved in gene expression regulation (mainly chromatin modification and transcription regulation) and those involved in neuronal communication (mainly synaptic communication and ion channel regulation) (42, 43). This suggests that dysregulation in gene expression and neuronal communication may significantly contribute to the genetic components underlying syndromic ASDs. We speculate that alterations in the genetic functions pertinent to expression regulation and neuronal communication may be fundamental contributors to the genetic components associated with severe-profound GDD/ID and multiple NCCs inherent in syndromic ASDs. Furthermore, neuronal communication between the craniofacial ectoderm and neural crest cells are vital for craniofacial patterning and morphogenesis during craniofacial development (44). We, therefore, speculate that alterations in these neuronal communication-related genes can cause disruption between the craniofacial ectoderm and neural crest cells, leading to a broad spectrum of craniofacial anomalies, among which BMs and HCAs are prominent phenotypic features. However, these speculations warrant further exploration through in-depth mechanistic experiments.

Additionally, previous research had demonstrated that the four phenotypic features—severe to profound GDD/ID, complicated NCC complexity, the presence of HCAs, and BMs—are strong indicators of rare monogenic neurodevelopmental disorders (24, 45). moreover, variants at exon-level have been recognized as the main cause of rare monogenic neurodevelopmental disorders (46). Given the close relationship between these elements, it is reasonable to infer that individuals with LF-ASDs exhibiting multiple phenotypic indicators associated with rare monogenic disorders may possess greater probabilities of harboring relevant exon-level variants, thereby facilitating a more straightforward diagnosis via trio-WES. However, LF-ASDs is a very complicated disorder with a high heterogeneity of genetic abnormalities including variants at exon-level and at out-exon-level (such as non-coding variation, epigenetics, and polygenic effects); given the technical limitation (exon-level sequencing only) of trio-WES, there is definitely a part of LF-ASDs patients cannot get an exact genetic diagnosis by using trio-WES alone because their genetic components may lie outside exon regions. Because of the lack of effective diagnostic tools (such as WGS and RNA-seq) applied in current study, we cannot determine whether the specific causes of those cases with negative trio-WES diagnosis in our study are non-coding variants, epigenetics, polygenic effects or other unknown genetic factors. Further analysis of these cases are needed to ascertain their specific etiologies and will be a focus of our future investigations.

Notably, the current study established a logistic regression model based on the four easily obtained phenotypic indicators and showed good calibration and discrimination with high accuracy and precision within both the training and validation LF-ASDs groups, revealing promising clinical applications. Moreover, considering the diagnostic yield (53.6%) and the number of identified variables (four) in the training cohort, in conjunction with the 10 events per variable, i.e., 10 EPV, the expected total number of cases in the training set should be over 75 (4 × 10 ÷ 0.536) (47). In actuality, the training cohort comprised 168 cases, which is substantially greater than 75, further affirming the reliability and robustness of the constructed nomogram.

However, the current study has several limitations. First, this study adopted a dual-center design, whereby the case sources for the training set (from a tertiary hospital) and the validation set (from a specialized LF-ASDs rehabilitation center) differed, potentially introducing selection bias. A multicenter study with a consistent case source (where all training and validation cases come from tertiary hospitals) are needed to further validate our predictive model. Second, our nomogram was constructed and validated within professional medical institutions, leaving its performance in primary medical institutions undetermined. Lastly, the study’s scope was not comprehensive enough, as it only considered ADHD and epilepsy as common NCCs in children with LF-ASDs. Further improvements, such as applying cutting-edge and Chinese version assessment tools to enable objective assessment of sleep and anxiety disorders in young children, allowing for the incorporation of such phenotypic variables into our predictive model, are required to further enhance the reliability of the model.

## Conclusion

We developed and validated an user-friendly nomogram based on common, objective, and easily obtained phenotypic indicators related to neurological conditions to predict the individualized diagnostic probability of trio-WES in children with LF-ASDs. This tool could assist affected children and their families in estimating their personalized diagnostic probability and selecting more suitable diagnostic strategies at the pre-diagnosis stage, ultimately reducing unnecessary financial expenditures. Additionally, this nomogram may enable pediatricians to identify children with LF-ASDs at high risk for relevant genetic factors at the early admission stage, facilitating more individualized patient management and subsequent genetic counseling.

## Data Availability

The original contributions presented in the study are included in the article/Supplementary material, further inquiries can be directed to the corresponding author.

## Footnotes

^1^ https://mutationtaster.org/

^2^PROVEAN; http://provean.jcvi.org/index.php

^3^Polyphen-2; http://genetics.bwh.harvard.edu/pph2/

^4^SIFT; https://sift.bii.a-star.edu.sg/

^5^CADD; https://cadd.gs.washington.edu/

^6^OMIM; https://omim.org/

^7^version 4.4.2, http://www.R-project.org/

^8^SFARI, http://gene.sfari.org/
